# Differences in Mammalian Target of Rapamycin Gene Expression in the Peripheral Blood and Articular Cartilages of Osteoarthritic Patients and Disease Activity

**DOI:** 10.1155/2013/461486

**Published:** 2013-06-25

**Authors:** Elena V. Tchetina, A. Robin Poole, Elena M. Zaitseva, Eugeniya P. Sharapova, Natalya G. Kashevarova, Elena A. Taskina, Liudmila I. Alekseeva, Liudmila A. Semyonova, Svetlana I. Glukhova, Alexandr N. Kuzin, Maxim A. Makarov, Sergey A. Makarov

**Affiliations:** ^1^Clinical Immunology Department, Research Institute of Rheumatology, Russian Academy of Medical Sciences, Moscow 115522, Russia; ^2^Department of Surgery, McGill University, Montreal, QC, Canada H3A OG4; ^3^Osteoarthritis Laboratory, Research Institute of Rheumatology, Russian Academy of Medical Sciences, Moscow 115522, Russia; ^4^Pathomorphology Department, Research Institute of Rheumatology, Russian Academy of Medical Sciences, Moscow 115522, Russia; ^5^Statistics Department, Research Institute of Rheumatology, Russian Academy of Medical Sciences, Moscow 115522, Russia; ^6^Forensic Medicine Service, Moscow City Health Department, Moscow 111020, Russia; ^7^Surgery Department, Research Institute of Rheumatology, Russian Academy of Medical Sciences, Moscow 115522, Russia

## Abstract

The gene expression of *mTOR*, autophagy-related *ULK1*, *caspase 3*, CDK-inhibitor *p21*, and *TNF* **α** was measured in the peripheral blood of osteoarthritic (OA) patients at different stages of the disease aiming to establish a gene expression profile that might indicate the activity of the disease and joint destruction. Whole blood of 65 OA outpatients, 27 end-stage OA patients, 27 healthy volunteers, and knee articular cartilages of 28 end-stage OA patients and 26 healthy subjects were examined. OA outpatients were subjected to clinical testing, ultrasonography, and radiographic and WOMAC scoring. Protein levels of p70-S6K, p21, and caspase 3 were quantified by ELISA. Gene expression was measured using real-time RT-PCR. Upregulation of *mTOR* gene expression was observed in PBMCs of 42 OA outpatients (“High *mTOR* expression subset”) and in PBMCs and articular cartilages of all end-stage OA patients. A positive correlation between *mTOR* gene expression in PBMCs and cartilage was observed in the end-stage OA patients. 23 OA outpatients in the “Low *mTOR* expression subset” exhibited significantly lower *mTOR* gene expression in PBMCs compared to healthy controls. These “Low *mTOR*” subset subjects experienced significantly more pain upon walking, and standing and increased total joint stiffness versus “High *mTOR*” subset, while the latter more often exhibited synovitis. The protein concentrations of p70-S6K, p21, and caspase 3 in PBMCs were significantly lower in the “Low” subset versus “High” subset and end-stage subjects. Increases in the expression of *mTOR* in PBMCs of OA patients are related to disease activity, being associated with synovitis more than with pain.

## 1. Introduction

Osteoarthritis (OA) is a systemic condition that can affect single or multiple joints and involves degenerative changes in the articular cartilage, remodeling of the subchondral bone, and limited synovial inflammation [1]. At present, the disease course is generally monitored by clinical and radiographic changes, which show poor sensitivity. Therefore, there is a need to identify new approaches in indicating disease activity.

Detection of gene expression changes measured in the whole blood is an emerging approach in OA research. Blood-based gene expression patterns recently obtained in transcriptome and microarray analyses appeared capable of distinguishing OA patients from control subjects [2, 3], already showing promising results. Moreover, the level of *IL-1*β** gene expression in peripheral monocytes has been proposed for OA patient stratification, as upregulation of *IL-1*β** was accompanied by increased pain and predicted higher risk of radiographic progression of the disease [4].

Recently evidence has been presented that disease manifestation is preceded by phenotypic modification (hypertrophy) of articular chondrocytes similar to that observed in fetal chondrocytes during their maturation in the growth plate [1, 5]. This was associated with the upregulation of genes involved in cartilage destruction and abnormal expression of regulatory proteins, such as growth and transcription factors, as well as apoptosis markers [6–8]. Other studies have reported that the majority of the identified genes involved in OA encode signal-transduction proteins [9].

Alteration in non-tissue-specific regulatory protein expression associated with disease manifestation may suggest differential gene expression in tissues other than cartilage, for example, blood. This is supported by the observation of modification in the expression of genes associated with fetal chondrocyte differentiation such as bone morphogenetic proteins 2, 4, and 6, as well as transcription factor *Runx2*, in the peripheral blood of OA patients [10].

Here we hypothesized that expression of genes associated with global cell survival and functioning, such as those involved in cell growth and proliferation, apoptosis, autophagy, and inflammation, measured from the whole blood of OA patients might point to the disease activity.

As extracellular matrix degradation in early OA is associated with chondrocyte hypertrophy [6], cessation of proliferative activity could involve changes in the expression of cyclin-dependent kinase (CDK) inhibitors, such as p21, whose overexpression is associated with the induction of genes expressed in different age-related disorders [11]. However, data on the activity of CDK inhibitors in chondrocytes of OA patients are inconclusive, as both activation [12, 13] and downregulation of p21 [14] have been reported in OA chondrocytes.

Alternatively, mTOR (mammalian target of rapamycin) is considered a key regulator of cell growth and proliferation [15], and its expression has been reported both in fetal chondrocytes in animal studies [16–18] and in human articular chondrocytes [19]. Moreover, treatment of mice with the mTOR inhibitor rapamycin or its analogs, has been recently shown to reduce the severity of experimental osteoarthritis [20, 21] and inflammatory arthritis [19, 22].

The effect of mTOR inhibition on cessation of growth is accompanied by the activation of autophagy [23], which has been observed both in OA articular chondrocytes [16, 20] and in peripheral blood cells [24]. This process occurs in lysosomes with membranes that contain proteins of the ULK (hATG) family and favors cellular survival [25].

Apoptosis represents a major cell death mechanism in eukaryotic cells. At present apoptotic activity in OA patients has been assessed only in articular chondrocytes [26, 27]. Some of these studies reported that cartilage destruction is accompanied by a significant increase in apoptotic activity [28, 29] while others observed only a few apoptotic cells in OA articular cartilage [30].

Although OA is not considered a classical inflammatory arthropathy because of the absence of neutrophils in the synovial fluid and the lack of marked systemic manifestations of inflammation, proinflammatory cytokines are involved in OA articular cartilage resorption [31, 32]. This is associated with increased expression of *IL-1* and *TNF*α** [32–34].

In the present study, we analyzed the expression of genes responsible for cell proliferation and growth (*mTOR*), regulation of cell cycle progression (*p21*), apoptosis (*caspase 3*), and autophagy (*ULK1*), as well as the proinflammatory cytokine *TNF*α**,in the whole blood and articular cartilage of knee OA patients at different stages of their disease. Our results suggest that differences in the expression of these genes might serve as an indicator of disease activity, symptoms, and knee joint destruction and provide new insights into OA pathobiology.

## 2. Materials and Methods

### 2.1. Ethics

The study protocol was approved by the Local Committee on the Ethics of Human Research and informed consent was obtained from all subjects.

### 2.2. Patients

*The Inclusion Criteria of the OA Patients and Control Subjects. *The control group consisted of 27 postmenopausal healthy females (average age 58.6 ± 8.3 years, range 42–74 years) free of any serious diseases recruited from the Moscow area. The control subjects were of comparable age to the OA outpatient group. The exclusion criteria for control subjects included any knee pain and crepitus, as well as low bone mineral density (BMD) (*T* score < –2.5 SD). The recruited controls were subjected to blood testing, including analyses of biochemical and hematological parameters, and densitometry of the lumbar spine and femur.

The OA outpatient group consisted of 47 unrelated postmenopausal Russian women with primary knee OA who had visited the outpatient clinic of the Institute of Rheumatology, at the Russian Academy of Medical Sciences between December 2007 and June 2009 (set 1). The average age of set 1 OA outpatients was 60.3 ± 7.1 years (range 47–74 years). These patients had radiological Kellgren & Lawrence (K&L) OA grades of II–IV. Set 2 consisted of 18 unrelated postmenopausal Russian women with primary knee OA who had visited the outpatient clinic of the Institute of Rheumatology, at the Russian Academy of Medical Sciences between February 2012 and July 2012. The average age of set 2 OA outpatients was 61.6 ± 8.3 years (range 47–74 years), with K&L OA grades between II-III.

All of the OA outpatients had moderate rates of knee pain according to VAS (40–70 mm) and normal BMD. For pain medication the following NSAID were used: meloxicam (15 mg/day), nimesulide (200 mg/day), or aceclofenac (200 mg/day) (Table 1). Patients were also treated with the chondroprotective agent chondroitin sulfate (1 g/day) with or without glucosamine sulfate (1 g/day).

We also examined the peripheral blood of 14 end-stage postmenopausal female OA patients undergoing knee joint replacement surgery aged 49 to 71 years (average age 56.6 ± 8.9 years) (set 1). In addition, we examined the peripheral blood of another 13 end-stage postmenopausal female OA patients undergoing knee joint replacement surgery aged 46 to 72 years (average age 59.3 ± 8.9 years) (set 2). Knee articular cartilage was also obtained from the same set 2 end-stage patients for the studies of *mTOR* gene expression correlation in the blood versus cartilage. All end-stage patients had OA radiological K&L grades of III or IV, experienced severe pain, and had walking problems (lameness).

All of the examined patients fulfilled the criteria of the American College of Rheumatology regarding OA [35].

*The Exclusion Criteria for OA Patients and Healthy Subjects.* The exclusion criteria were rheumatoid arthritis; secondary arthritis associated with reactive arthritis, systemic inflammatory joint diseases, gout, pseudogout, Padgett's disease, intraarticular fractures, ochronosis, acromegaly, hemochromatosis, Wilson disease, primary synovial chondromatosis, chondrocalcinosis, aseptic necrosis of femoral or tibia condyles, orany type of knee surgery; and any abnormalities of bone metabolism including diabetes mellitus; renal diseases; thyroid, parathyroid, or other endocrinological diseases; uncontrolled arterial hypertension; instable angina; vascular insufficiency; gastric or duodenal ulcer; bleeding; or thrombophlebitis. Women who had taken drugs such as estrogen, progesterone, glucocorticoids, bisphosphonates, and alfacalcidol were not included in the study.

### 2.3. Cartilage

Human femoral condylar cartilage was obtained at total knee arthroplasty from 15 patients (4 men, mean age 64.5 ± 14.9 years; range 44 to 79 years and 11 women, mean age 57.8 ± 7.8 years; range 40 to 71 years) (set 1) and another 13 end-stage postmenopausal female patients aged 46 to 72 years (average age 59.3 ± 8.9 years) (set 2) with OA diagnosed according to the criteria of the American College of Rheumatology [35].

Human articular cartilage from 14 healthy individuals (9 men, mean age 45.0 ± 5.1 years; range 39 to 51 years and 5 women, mean age 31.5 ± 3.5 years; range 29 to 34 years) (set 1 controls) and another 12 healthy individuals (10 men, mean age 36.0 ± 7.7 years; range 25 to 45 years and 2 women, mean age 35.0 ± 2.1 years; range 34 to 37 years) (set 2 controls) was obtained in less than 12 hours post mortem at autopsy from the femoral condylar surfaces of the knee that articulate with the patella.

No patient had received chemotherapy or had diabetes or lower limb vascular insufficiency. In articular cartilage studies, we used specimens of both genders due to limitations in specimen availability.

In each knee, full-depth cartilage (to subchondral bone) was removed. The cartilage was analyzed for histology and gene expression. Cartilage samples from healthy patients contained normal cartilage, as revealed by a Mankin grade of 1-2. The degree of degeneration in the OA cartilage samples was 7.8 ± 2.5; range 5 to 12 [36, 37].

### 2.4. Clinical Testing

OA grade and osteophyte presence were determined by the analysis of the weight-bearing anteroposterior radiographs of the knees. These were scored on a five-point scale (0–4) according to Kellgren & Lawrence [38].

The WOMAC (Western Ontario and McMaster Universities osteoarthritis index) visual analogue scale was used to assess pain, stiffness, and physical function [39].

Synovitis registered in the medical history was diagnosed by ultrasonography and by joint effusion.

### 2.5. Ultrasound Examination for Synovitis

Knees were examined using a Voluson station (GE Medical Systems, Kretztechnik GmbH & Co. OHG, Zipf, Austria) with a multifrequency linear 4–13 MHz probe. The presence of synovitis was assessed according to the EULAR guidelines [40].

### 2.6. BMD Measurement

BMD at the lumbar spine (L1–L4), femoral neck, and total femur was measured by dual-energy X-ray absorptiometry using a QDR-4500w instrument (Hologic, USA). According to the criteria recommended by the World Health Organization [41], a *T* score of < –2.5 SD, all subjects examined in this study were diagnosed as free of osteoporosis.

### 2.7. Peripheral Blood Fractionation

Peripheral blood (10 mL) was collected in Vacutainer tubes containing ethylenediaminetetraacetic acid (EDTA) (BDH, England). The blood samples were taken in a standardized manner in the morning (between 07:00 a.m. and 09:00 a.m.). Whole blood fractionation was performed using a Ficoll density gradient. Upon centrifugation, blood samples were separated into plasma enriched with thrombocytes, peripheral blood mononuclear cells (PBMCs) located in the interphase, and a pellet containing granulocytes on top of red blood cells [42]. Every cell fraction was collected and washed twice in phosphate-buffered saline (PBS). Erythrocytes were lysed using hypotonic buffer (1.6 mM EDTA, 10 mM KHCO_3_, and 153 mM NH_4_Cl, pH 7.4), which was added at a 3 : 1 volume ratio. The obtained cell fractions were frozen and kept at −70°C prior to protein extraction or were used immediately for RNA isolation.

### 2.8. Quantification of p70-S6K, p21, and Caspase 3 Protein Levels

Concentrations of total p70-S6K (KHO0571), phospho-p70-S6K (KHO0581), p21WAF1/Cip1 (KHO5421), and active caspase 3 (KHO1091) were determined in isolated PBMCs using commercially available enzyme-linked immunosorbent assay (ELISA) kits (Invitrogen, Camarillo, CA, USA) according to the manufacturer's instructions. For mTOR protein expression, we evaluated levels of p70-S6K, an mTOR direct target for phosphorylation, which is usually used as an mTOR readout [43–45], as mTOR ELISA kits are not available in Russia.

Results were expressed per *µ*g of protein measured in PBMC lysates. PBMC lysates were obtained using Cell Extraction Buffer containing 10 mM Tris, pH 7.4, 100 mM NaCl, 1 mM EDTA, 1 mM EGTA, 1 mM NaF, 20 mM Na_4_P_2_O_7_, 20 mM Na_3_VO_4_, 1% Triton X-100, 10% glycerol, 0.1% SDS, and 0.5% deoxycholate (Invitrogen, Camarillo, CA, USA) supplemented with Protease Inhibitor Cocktail (Sigma-Aldrich, Inc., St. Louis, USA) and 1 mM PMSF (Sigma-Aldrich, Inc., St. Louis, USA) according to the manufacturer's instructions. Total protein concentration in cell lysates was quantified by the Bradford method [46].

### 2.9. Total RNA Isolation and Reverse Transcriptase (RT) Reaction

For detection of gene expression total RNA was isolated from 100 *μ*L of whole blood immediately, after withdrawal, from serum, from erythrocyte lysate, or from 10^7^ freshly isolated cells using Ribo-zol-A kit (InterLabService, Moscow, Russia) in accordance with the manufacturer's recommendations. Total RNA had an *A*_260/290_ > 1.9. Total RNA was also isolated from fresh knee articular cartilage using TRIzol reagent according to the manufacturer's recommendations (Invitrogen, Carlsbad, CA, USA). The RT reaction was performed using a Reverta kit containing M-MLV reverse transcriptase, random hexanucleotide primers, and total RNA according to the manufacturer's recommendations (InterLabService, Moscow, Russia).

### 2.10. Real-Time Quantitative PCR

Premade primers and probes for the TaqMan assay (Applied Biosystems, Foster City, CA, USA) of human genes used in this study were: *mTOR* (Hs00234522_m1), *Unc-51-like kinase 1* (*ULK1)* (Hs00177504_m1), *p21WAF1/Cip1* (*p21*) (Hs00355782_m1), *caspase 3* (Hs00263337_m1), *TNF*α** (Hs00174128_m1), *COL10A1* (Hs00166657_m1), *MMP-13* (Hs00233992_m1), and *MMP-9* (Hs00234579_m1). *β*-Actin was used as an endogenous control.

Quantification of gene expression was conducted using a 7300 Real-Time PCR System (Applied Biosystems, Foster City, CA, USA). A volume of 1 *μ*L of RT product was subjected to real-time PCR in a 15 *μ*L total reaction mixture containing 7.5 *μ*L of TaqMan Universal PCR Master Mix (Applied Biosystems), 900 nM sense and antisense primers, 50 nM probe, and template cDNA. After a single step of 50°C for 2 min and initial activation at 95°C for 10 min, reaction mixtures were subjected to 40 amplification cycles (15 s at 95°C for denaturation and 1 min of annealing and extension at 60°C).

Relative mRNA expression was determined using the delta-delta *C*_*T*_ method, as detailed by the manufacturer's guidelines (Applied Biosystems) [47]. The delta *C*_*T*_ value was calculated by subtracting the *C*_*T*_ value for the housekeeping gene *β*-actin from the *C*_*T*_ value for each sample. A delta-delta *C*_*T*_ value was then calculated by subtracting the delta *C*_*T*_ value of the control (each healthy patient) from the delta *C*_*T*_ value of each OA patient. Each PCR was performed in duplicate. Three “no template” controls were consistently negative for each reaction.

For subset division, we initially measured *mTOR* gene expression in relation to *β*-actin expression in healthy subjects. Then, we calculated *mTOR* gene expression in each of 27 healthy subjects using each of the same 27 healthy subjects as a calibrator. The average of all the obtained fold change values related to each healthy subject was considered the relative individual gene expression for each healthy control subject. We observed normal distributions for these relative gene expression values (1.04 ± 0.21 in the case of *mTOR*) (Supplementary Table 3 in Supplementary Material available online at http://dx.doi.org/10.1155/2013/461486). In further gene expressions testing, eight cDNAs from the healthy control subjects were placed on each PCR plate to reproduce initial relative gene expression results in the corresponding control subjects. The relative gene expression of each OA patient was compared to the pool of eight healthy controls using 7300 Sequence Detection Software Version 1.3.1 (Applied Biosystems) and the remainder 19 healthy controls using *C*_T_ values obtained in the preliminary studies. The average of all the obtained fold change values related to each OA subject was considered the relative individual gene expression for each OA subject. The subjects whose *mTOR* relative gene expression was significantly lower than that of controls (*P* < 0.05) were registered to the “Low” subset; otherwise, they were registered to the “High” subset.

### 2.11. Statistical Analysis

A Kolmogorov-Smirnov normality test showed that the data were distributed according to a Gaussian distributive curve. Therefore, for statistical evaluations, Pearson's rank correlations and unpaired Student's *t*-test were used for comparisons between the control subjects and OA patient subsets. Quantitative data were expressed as the means ± SD. Differences in gene expression between the control group and the OA patient subsets were also tested using a two-way analysis of variance (ANOVA), followed by Scheffe's post-hoc test to confirm the results. Levene's test was used to assess a difference in variance between groups. Non-normally distributed data was expressed as median (quartiles) and Mann-Whitney *U* test was applied. To compare percentages, a two-tailed *Z*-test for percentages was applied. Statistica 6 Software (StatSoft, Tulsa, OK, USA) was used for all statistical analyses. *P* values less than 0.05 were considered significant.

## 3. Results

### 3.1. Clinical Parameters of OA Outpatients

Analysis of the demographic and clinical characteristics of 47 OA outpatients (set 1) revealed that the K&L OA grades of the examined subjects varied from II to IV (grade II, 29 patients; grade III, 12 patients; and grade IV, 6 patients). The average disease duration was 11.6 years (range 1–30 years). The majority of patients exhibited Heberden's nodes and an increased BMI (range 20.5–45.9). All the patients had normal bone mineral density (BMD). WOMAC scoring indicated moderate rates of knee pain (below 65 mm according to VAS) in these OA outpatients. The average joint stiffness was 83.4 (range 20–126). Synovitis at the knee joint was detected in half (48.9%) of the OA outpatients.

### 3.2. Whole Blood Gene Expression

**Figure 1:**
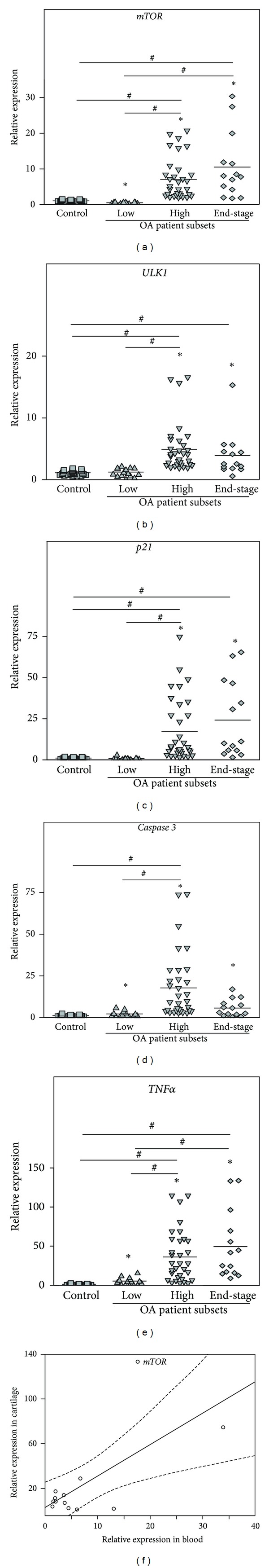
Relative expression of the genes *mTOR* (a), *ULK1* (b), *p21* (c), *caspase 3* (d), and *TNF*α** (e) with reference to *β*-actin determined by real-time PCR analyses in the whole blood of “Low *mTOR* subset” (*n* = 15), “High *mTOR* subset” (*n* = 32), and end-stage OA patients (*n* = 14) (set 1) compared with healthy controls (*n* = 27). (f) A relationship between *mTOR* gene expression measured in the blood or articular cartilage from end-stage OA patients (*n* = 13, set 2). Controls are shown as 1.0 as required for relative quantification with the real-time PCR protocol. Asterisks (∗) indicate significant differences from the control in pairwise comparisons (Student's unpaired *t*-test). Number signs (#) show significant differences in multigroup comparisons using two-way ANOVA followed by the Scheffe's post-hoc test.

Examination of gene expression in the whole blood of 47 OA outpatients (set 1) revealed that *mTOR* was significantly downregulated in 15 patients compared to the healthy controls, while the remaining 32 subjects exhibited *mTOR* gene upregulation (Figure 1(a)). As cellular metabolism has been shown to be dramatically affected by the level of *mTOR* expression [15, 17, 18], the examined OA outpatients were divided into 2 subsets: a “Low *mTOR* expression subset” (15 patients) and a “High *mTOR* expression subset” (32 patients). These subsets also demonstrated differences in clinical characteristics as presented in Table 1.

Statistical analysis of the *mTOR* gene expression data for all of the OA outpatients did not result in a normal distribution (Kolmogorov-Smirnov test (K-S) *d* = 0.23, *P* < 0.05). Normal distribution of the *mTOR* gene expression values was observed in both the “Low *mTOR* expression subset” (K-S *d* = 0.163, *P* > 0.2) and the “High *mTOR* expression subset” (K-S *d* = 0.187, *P* < 0.2) of OA patients. The data for relative *mTOR* gene expression are presented in Supplementary Table 3.

Stratification of OA outpatients by disease stage did not show significant differences in the relative expression of *mTOR*, values of which were 5.7 ± 7.8 for grade II (*n* = 29),  5.1 ± 7.0 for grade III (*n* = 12), and 3.1 ± 2.7 for grade IV outpatients (*n* = 6).

Peripheral blood from the same subsets of OA outpatients was also examined for the expression of the autophagy marker *ULK1*, the regulator of cell cycle progression, a cyclin-dependent kinase inhibitor *p21*, the apoptosis indicator *caspase 3*, and the proinflammatory cytokine *TNF*α**. These analyses demonstrated that the “Low *mTOR* expression subset” OA outpatients exhibited significant upregulation of *caspase 3* and *TNF*α** genes, while *ULK1 *and *p21* expression remained similar to that in healthy individuals (Figures 1(b)–1(e)).

In contrast, the “High *mTOR* expression subset” OA outpatients exhibited significant upregulation of all the examined genes in comparison to healthy controls (Figure 1). Gene expression studies in the end-stage OA patients undergoing joint replacement surgery also demonstrated that all of the examined genes were overexpressed in their whole blood in comparison to healthy controls. A two-way analysis of variance (ANOVA) followed by Scheffe's post-hoc test of gene expression in the same control group and the OA patient subsets showed essentially similar results (Figure 1).

### 3.3. Association of Gene Expression with Peripheral Blood Mononuclear Cells (PBMCs)

**Figure 2:**
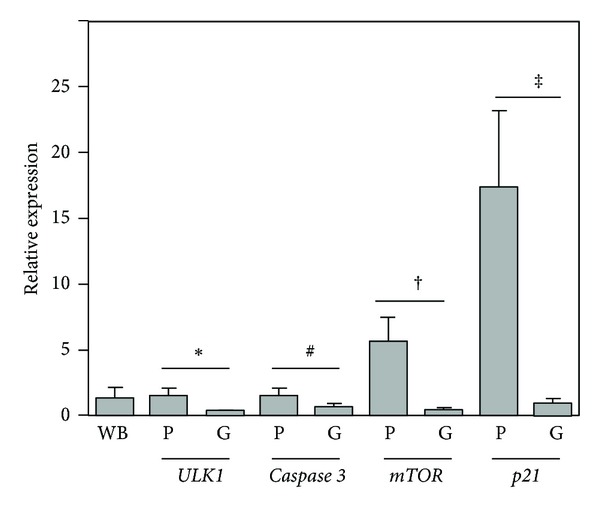
Relative expression of *mTOR*, *ULK1*, *p21*, and *caspase 3* genes by real-time PCR with reference to *β*-actin in PBMCs (P) or granulocytes (G) compared with whole blood (WB) specimen of OA outpatients (*n* = 12). The control is shown as 1.0 as required for relative quantification with the real-time PCR protocol. The following symbols: ∗, #, †, and ‡ indicate significant differences in gene expression between PBMCs and granulocytes (Student's unpaired *t*-test).

The cellular origin of tested RNAs was confirmed by the examination of gene expression in cellular and noncellular elements of the whole blood. There was no expression of the examined genes in serum, erythrocyte, or thrombocyte fractions. In contrast, significantly higher expression of the examined genes was revealed in the PBMC fraction in comparison to isolated granulocytes in both the OA patients (Figure 2) and healthy subjects (data not shown).

### 3.4. Protein Levels of Total- and Phospho-p70-S6K, p21, and Active Caspase 3 in Isolated PBMCs

**Figure 3:**
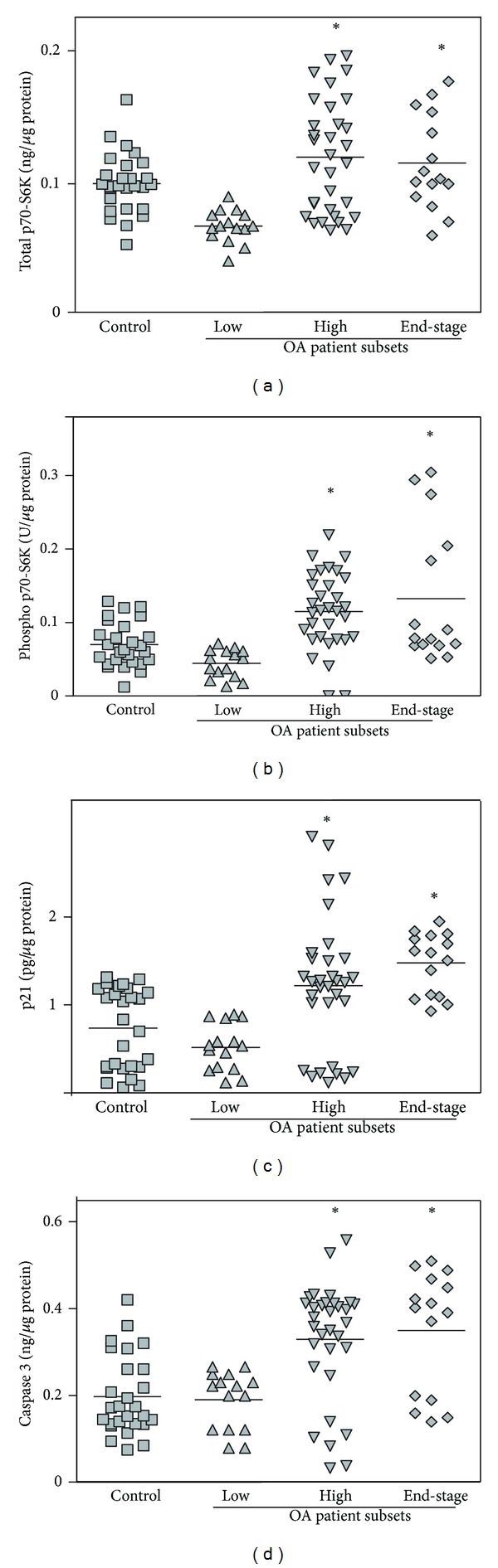
Protein concentrations of total p70-S6K (a), phospho-p70-S6K (b), p21 (c), and active caspase 3 (d) measured by ELISA in PBMCs from the “Low *mTOR* subset” (*n* = 15), the “High *mTOR* subset” (*n* = 32), and end-stage (*n* = 14) OA patients compared with control subjects (*n* = 27) (set 1). Asterisks indicate significant differences from the healthy control patients (Student's unpaired *t*-test).

To further investigate the clinical significance of *mTOR, p21*, and *caspase 3* relative gene expression in the whole blood of OA outpatients and end-stage OA subjects, we analyzed the protein levels of total- and phospho-p70-S6K serine/threonine kinase (a direct target for phosphorylation by mTOR [43–45]), p21, and active caspase 3 in the PBMC fraction. The “Low *mTOR* expression subset” OA outpatients possessed significantly lower total- and phospho-p70-S6K, p21, and caspase 3 protein concentrations in PBMCs compared to the “High *mTOR* expression subset” of OA outpatients and the end-stage OA subjects (Figures 3(a)–3(d)). When the amount of the examined proteins was evaluated compared to that in healthy subjects, we observed that the “Low *mTOR* subset” of OA outpatients possessed significantly lower total- and phospho-p70-S6K proteins, while p21 and caspase 3 levels were not significantly different. In contrast, protein concentrations of all of the examined genes in the “High *mTOR* subset” of OA outpatients and in the end-stage OA subjects significantly exceeded those in healthy individuals (Figures 3(a)–3(d)).

### 3.5. Clinical Characteristics of “Low *mTOR*” and “High *mTOR*” Subsets of OA Outpatients

The outpatients in the designated subsets exhibited important differences in the manifestation of clinical traits (Table 1). The “Low *mTOR*” subset outpatients experienced significantly more pain upon walking and standing and increased total joint stiffness compared to the “High *mTOR*” OA subjects. They also were more often diagnosed Heberden's nodes, although not to a level of statistical significance.

In contrast, in the “High *mTOR*” subset of OA outpatients, we observed a significantly higher incidence of synovitis and reduced total femur BMD compared to the “Low *mTOR*” OA subjects, as well as an increased severity of night pain and lower BMD at the femoral neck, although these differences were not statistically significant.

The analysis of K&L radiological stage distribution among the OA outpatients revealed the higher relative numbers of stage II patients in the “High *mTOR*” subset compared to the “Low *mTOR*” subset. In contrast, a higher relative number of stage III patients belonged to the “Low *mTOR*” subset versus the “High *mTOR*” OA outpatients. However, these differences were not statistically significant.

The examination of the medication used for treatment revealed that the majority of OA outpatients (31 out of 47) in both subsets were treated by meloxicam, while 11 out of 47 outpatients received nimesulide. Only two patients from the subset “High *mTOR*” were treated with aceclofenac, while 3 patients from the subset “Low *mTOR*” did not require any anti-inflammatory medication. Chondroitin sulfate with or without glucosamine was used as a chondroprotective agent for OA outpatients of both subsets (Table 1). However, significantly higher number of the “High *mTOR*” subset outpatients were treated with chondroitin sulfate plus glucosamine compared to the “Low *mTOR*” OA outpatients.

The analysis of bivariate correlations using Pearson's correlation coefficients on expression of the examined genes showed that they were positively correlated to each other in the designated subsets of OA patients (Table 2). In contrast, healthy subjects showed a negative correlation between *mTOR* and *TNF*α** gene expression, while *ULK1 *and *caspase 3* expression was positively correlated with *TNF*α** expression.

The expression of the examined genes also correlated with clinical traits. Thus, the “Low *mTOR* subset” outpatients showed a positive correlation between *caspase 3* gene expression and spine and femur BMD, while a negative correlation was observed between the expression of *mTOR* and *caspase 3* genes and WOMAC indices (Table 2).

The “High *mTOR*” subset outpatients also demonstrated a positive correlation between spine and femur BMD and *ULK1* and *mTOR *expression. *TNF*α** gene expression was positively correlated with ESR while *mTOR* expression was positively correlated with BMI (Table 2).

### 3.6. Examination of Gene Expression in the Articular Cartilage of End-Stage OA Patients

**Figure 4:**
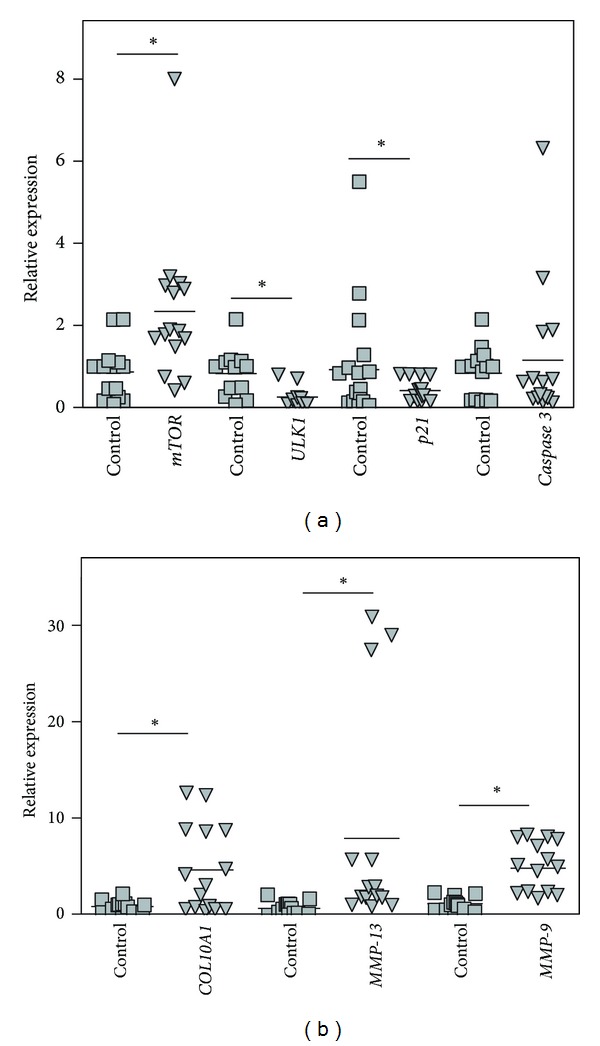
Relative expression of *mTOR*, *ULK1*, *p21*, *caspase 3* (a), *COL10A1*, *MMP-13*, and *MMP-9* (b) genes compared between healthy cartilages (*n* = 14, set 1) and osteoarthritic cartilages (*n* = 15, set 1) as determined by real-time PCR analyses with reference to *β-*actin. Controls are shown as 1.0 as required for relative quantification with the real-time PCR protocol. Asterisks (∗) indicate significant differences from the control (Student's unpaired *t*-test).

Articular cartilage degradation in the 15 end-stage OA patients (set 1) at arthroplasty was associated with significantly higher levels of matrix metalloproteinase *MMP-13* and *MMP-9,* as well as type X collagen (*COL10A1*) gene expression in cartilage compared to the corresponding levels in healthy subjects (Figure 4). This was accompanied by a significant upregulation of *mTOR* expression and downregulation of *ULK1* and *p21* in comparison to healthy controls (Figure 4). At the same time, no significant changes in *caspase 3 *gene expression in these OA patients versus healthy subjects were observed.

### 3.7. Correlation of Peripheral Blood Mononuclear Cell (PBMC) and Articular Cartilage Chondrocyte Gene Expression

Plotting of *mTOR* gene expression in PBMC versus that in the articular cartilage of the same 13 end-stage OA patients (set 2) revealed a significant correlation (Pearson's correlation coefficient *r* = 0.687; *P* = 0.01) (Figure 1(f)). Without the outliers Pearson's correlation coefficient came to *r* = 0.93; *P* = 0.0001 (Supplementary Figure 5).

### 3.8. Reproducibility of Data

To determine whether these results were reproducible, the expressions of the same genes were reexamined in other patients. Thus we analyzed the blood of another group of 18 OA outpatients (set 2) and another 13 end-stage OA patients (set 2); in addition we examined the articular cartilage of another 13 end-stage OA patients (set 2) compared to another set of healthy controls (set 2 controls, *n* = 12). Essentially similar results were obtained (Supplementary, Results, Figures  6 and 7).

## 4. Discussion

As repair potential of adult articular cartilage is very limited [1], informative indicators of OA disease activity are of importance, although OA is not classified as active or inactive. However, the rate of the disease progression is variable between individuals. Moreover, progression may be inconsistent in the same individual over time and joint degradation may occur intermittently [48–51]. Therefore, the distinction between disease and non-disease is not evident in OA [52]. The clinical definition of OA is based on a combination of symptoms and radiological findings, in which the correlation is weak, as patients with radiological OA may have no symptoms, while classical symptoms of OA may be accompanied by the absence of structural changes [53]. An approach involving registration of gene expression, which we applied in the present paper, offers the potential to better characterize disease activity and to distinguish between active versus inactive disease.

In this study, we demonstrate the value of an assessment of non-tissue-specific regulatory gene expression in the whole blood of OA patients for evaluating disease activity. We show that elevated *mTOR* gene expression in the PBMCs of a subset of OA patients with less-advanced disease might point to articular cartilage degradation because increased expression of this gene is seen in both peripheral blood and articular cartilage in end-stage OA patients requiring knee joint replacement. In addition, a positive correlation between *mTOR* gene expression in the blood and articular cartilage in the same end-stage OA patients suggests that upregulation of *mTOR* gene expression in the PBMCs might occur concomitantly with increased articular cartilage destruction.

Additionally, higher expression of the proinflammatory cytokine *TNF*α** and the significantly higher incidence of synovitis, which was observed in the “High *mTOR* expression subset” of OA outpatients, as well as a positive correlation between *mTOR* and *TNF*α** gene expression in all OA outpatient subsets but not in healthy subjects, would suggest an important contribution of inflammation to disease activity. This has also been suggested previously [4, 54–61]. The lower activity of synovitis in the “Low *mTOR*” subset OA outpatients is supported by the lower requirement for anti-inflammatory therapy as some patients could do without such medication.

At the same time examination of *mTOR* expression in the blood of postmenopausal OA women should be accompanied by a careful assessment of BMD indices. This became apparent in our other studies of a significant downregulation of *mTOR* gene expression in the blood of the postmenopausal osteoporotic patients ([62], Tchetina et al., paper in preparation).

In some studies, a positive association of knee pain with articular cartilage destruction has been observed in OA patients [63, 64]; however, we noticed that, although suffering significantly more pain, OA outpatients with low gene expression of *mTOR* were not observed among the end-stage OA patients undergoing joint replacement. Therefore, pain might be associated in these patients with periarticular tissue inflammation [65]. This is also supported by the observation that the subset “Low *mTOR*” of OA outpatients experienced increased pain upon joint function (walking or standing) but not at rest or at night. The limited value of knee pain in determining disease activity and progression has also been noted previously [66–68].

However, grade IV OA outpatients could become candidates for joint replacement and be considered end-stage patients if their pain was to exceed 70 cm according to the VAS, as severe pain usually leads to total joint replacement [69]. This could be accompanied by an increase in *mTOR* and proinflammatory cytokine gene expression. Therefore, the progression in disease in OA, which might be associated more with inflammation/synovitis than pain, could be monitored in the peripheral blood by *mTOR* gene expression level.

As the end-stage OA patients exhibited increased *mTOR* gene expression both in the PBMC and the articular cartilage, upregulation of this gene might designate those OA patients, which are more prone to joint replacement. Therefore, upregulation of *mTOR* gene expression might indicate the type of the OA disease activity associated with advanced cartilage destruction and synovitis. In contrast, *mTOR* downregulation might represent disease activity associated with reduced cartilage degeneration but with joint dysfunction resulting in increased joint pain and stiffness. The association of synovitis with night pain in the “High *mTOR*” group and pain on walking and standing in the “Low *mTOR*”set requires much studying to better understand these differences.

Although *mTOR* gene expression level might be indicative of OA activity, the assessment of gene expression alterations in markers for autophagy (*ULK1*), apoptosis (*caspase 3*), regulation of cell cycle progression (*p21*), and inflammation (*TNF*α**) is also important. As these genes are responsible for global cell survival and function, they could collectively represent a “metabolic signature” of the OA patient, indicating overall gene expression disturbances associated with and reflective of the disease activity.

Essentially, the same pattern of *mTOR, ULK1, p21*, and *caspase 3* gene expression was observed in the blood of the end-stage OA patients and the “High *mTOR*” subset of OA outpatients with less-advanced disease. However, the pattern of expression of the examined in our study genes was not always similar in the cartilage and blood of end-stage OA patients. This might result from differences in responses of chondrocytes versus white blood cells associated with the disease. *mTOR* reciprocally downregulates autophagy [70], as seen by the decrease of *ULK 1* expression in the cartilage of the end-stage OA patients in this and other studies [20]. The chondrocyte hypertrophy associated with OA and evidenced by the upregulation of *COL10A1, MMP-13*, and *MMP-9* gene expression in the examined OA cartilage has also been observed previously [71–75].

The results of our exploratory, correlative study suggest that upregulation of *mTOR *gene expression in the whole blood of OA outpatients is accompanied by increased synovial inflammation and might be associated with increased and possibly accelerated joint destruction later in the disease. Moreover, the assessment of *mTOR, ULK1, p21, caspase 3*, and *TNF*α** gene expression in the blood of OA patients could provide a patient's “metabolic signature” associated with the disease, which might be of use in elucidating the clinical efficacy of OA treatment and contribute to our understanding of the mechanisms underlying OA therapy.

There are limitations to the present study. Because of the relatively small size of the cohorts that have been studied, and consequently underpowering of the study, there is clearly a need for this investigation to be repeated in much larger cohorts to avoid the influence of possible confounders. Yet the fact that these findings were confirmed in a second, albeit even smaller population, holds promise for the value of these initial observations: these also require examination in a longitudinal investigation to examine their potential prognostic value.

## 5. Conclusions

Increased expression of *mTOR* in PBMCs of OA patients is related to the presence of synovitis and is seen in all patients requiring joint replacement. Those patients with low expression of *mTOR* experienced more pain on walking and standing and increased joint stiffness but were not among those with end-stage disease requiring arthroplasty. These analyses may be of value in better characterizing disease activity and cartilage degeneration in patients with knee OA.

## Supplementary Material

Supplementary Material section contains details on mTOR gene expression in the examined subjects as well as a description of gene and protein expression analyses in another set of 18 OA outpatients and 13 end-stage OA patients aiming to examine reproducibility of the data presented in the Results section.

## Figures and Tables

**Table 1:** Demographic and clinical characteristics of the outpatients with knee OA.

Set 1 patients	“Low *mTOR*” subset (*n* = 15)	“High *mTOR*” subset (*n* = 32)	*P* (Student's unpaired *t*-test)
Age, years	60.7 ± 6.7	60.3 ± 6.9	0.86
Disease duration, years	12.0 ± 8.8	11.2 ± 10.3	0.80
BMI, kg/m^2^	35.6 ± 5.4	32.3 ± 5.4	0.06
Menopause, years	11.4 ± 6.4	12.3 ± 7.2	0.69
Average K&L radiological stage			
II	46.6% (7/15)	68% (22/32)	0.15
III	40% (6/15)	18.7% (6/15)	0.16
IV	13.4% (2/15)	12.5% (4/32)	0.93
ESR, mm/h	13.1 ± 8.8	11.7 ± 6.6	0.55
Total WOMAC, mm	1202 ± 285	1102 ± 309	0.29
Pain on descending	64.6 ± 15.7	58.8 ± 17.7	0.28
Pain on ascending	62.1 ± 16.1	57.0 ± 17.9	0.35
Pain on move onset	51.8 ± 12.3	48.9 ± 22.6	0.64
Pain at rest	35.0 ± 20.3	31.8 ± 15.8	0.55
Pain on walking	59.2 ± 12.5	49.6 ± 10.5	**0.009**
Pain on standing	62.5 ± 13.0	50.8 ± 16.4	**0.02**
Pain at night	28.8 ± 19.4	35.6 ± 23.4	0.34
Total pain	364.2 ± 82.0	332.4 ± 93.7	0.26
Total stiffness	101.3 ± 25.7	75.0 ± 27.0	**0.003**
Total physical function	737.4 ± 217.6	695.0 ± 229.8	0.55
Heberden's nodes, %	80.0 (12/15)	59.0 (19/32)	0.08
Bouchard's nodes, %	27.0 (4/15)	18.7 (6/32)	0.26
Synovitis, %	20 (3/15)	62.5 (20/32)	**0.004**
BMD, g/cm^2^:			
Lumbar spine (L1–L4)	0.916 ± 0.1	0.960 ± 0.1	0.35
Femoral neck	0.843 ± 0.1	0.795 ± 0.1	0.16
Total femur	0.981 ± 0.1	0.884 ± 0.1	**0.01**
Anti-inflammatory treatment (%):			
Meloxicam	53 (8/15)	72 (23/32)	0.20
Nimesulide	27 (4/15)	22 (7/32)	0.70
Aceclofenac	0	6 (2/32)	—
None	20 (3/15)	0	—
Chondroprotective agents (%):			
Chondroitin sulfate	60 (9/15)	22 (7/32)	**0.01**
Chondroitin sulfate + glucosamine sulphate	40 (6/15)	78 (25/32)	**0.01**

Values given are mean ± SD. BMI: body mass index; K&L: Kellgren-Lawrence; ESR: erythrocyte sedimentation rate; WOMAC: Western Ontario and McMaster Universities osteoarthritis index; BMD: bone mineral density.

**Table 2:** Correlation coefficients (Pearson's) and their significance (*P*; Student's unpaired *t*-test) are shown for the expression of *mTOR, ULK1, p21,  caspase 3,* and *TNF*α** genes in OA patients and healthy subjects in relation to each other and clinical traits.

Set 1 patients	*mTOR *	*ULK1 *	*Caspase 3 *	*TNF*α**
**“**Low *mTOR*” subset OA patients (*n* = 15)				
*Caspase 3 *				0.645 *P* = 0.009
Pain on walking	−0.568 *P* = 0.02			
Pain on standing	−0.701 *P* = 0.004			
Total physical function			−0.674 *P* = 0.007	
Total WOMAC			−0.633 *P* = 0.007	
BMD L1–L4			0.544 *P* = 0.03	
BMD total femur			0.580 *P* = 0.02	

“High *mTOR*” subset OA patients (*n* = 32)				
* mTOR *		0.369 *P* = 0.03	0.408 *P* = 0.02	0.691 *P* < 0.001
*P21 *	0.360 *P* = 0.04	0.683 *P* < 0.001		0.354 *P* = 0.04
* Caspase 3*				0.502 *P* = 0.003
BMI	0.432 *P* = 0.01			
BMD L1–L4		0.454 *P* = 0.009		
BMD femoral neck	0.439 *P* = 0.01			
ESR				0.442 *P* = 0.01

End-stage OA patients (*n* = 14)				
* mTOR *				0.549 *P* = 0.04
* ULK1 *				0.813 *P* < 0.001
*P21 *		0.770 *P* = 0.001		0.756 *P* = 0.02

Healthy subjects				
*TNF*α**	−0.560 *P* = 0.03	0.639 *P* = 0.01	0.556 *P* = 0.03	

Only significant data are presented. ESR: erythrocyte sedimentation rate; WOMAC: Western Ontario and McMaster Universities osteoarthritis index; BMD: bone mineral density; BMI: body mass index.
