# Supplementary material for: Differences in Mammalian Target of Rapamycin Gene Expression in the Peripheral Blood and Articular Cartilages of Osteoarthritic Patients and Disease Activity
Source: Arthritis. 2013 Jun 25;2013:461486. doi: 10.1155/2013/461486 (PMC3707211; doi:10.1155/2013/461486)
Supplement: Supplementary file 1 — Supplementary Material section contains details on mTOR gene expression in the examined subjects as well as a description of gene and protein expression analyses in another set of 18 OA outpatients and 13 end-stage OA patients aiming to examine reproducibility of the data presented in the Results section. [file 461486.f1.pdf]

SUPPLEMENTARY MATERIAL for the manuscript:

Differences in *mTOR* (mammalian target of rapamycin) gene expression in the peripheral blood and articular cartilages of osteoarthritic patients and disease activity

by Elena V Tchetina et al.

Supplementary, Table 3: Relative mTOR gene expression in healthy control subjects (n=27), OA patients **(set 1)** ("LOW *mTOR* "subset, n=15; "HIGH *mTOR"* subset, n=32; END-STAGE patients, n=14), and OA patients **(set 2)** ("LOW *mTOR*" subset, n=8; "HIGH *mTOR*" subset", n=10; END-STAGE patients, n=13).

Healthy		mTOR relative gene expression
	1	1,08783
	2	1,29558
	3	1,04163
	4	0,94523
	5	0,96338
	6	1,2323
	7	1,01318
	8	1,08049
	9	0,93539
	10	1,35818
	11	0,64667
	12	0,96004
	13	0,99386

	14	1,09445
	15	1,37059
	16	1,22704
	17	0,81389
	18	1,21152
	19	0,81676
	20	1,26097
	21	0,97928
	22	1,3057
	23	0,88887
	24	0,8253
	25	0,79016
	26	0,7135
	27	1,3057
Subset "LOW *mTOR*" (set 1)		
	1	0,07389
	2	0,33365
	3	0,38008
	4	0,44799
	5	0,40175

	6	0,79174
	7	0,74165
	8	0,68741
	9	0,46235
	10	0,8302
	11	0,24414
	12	0,42502
	13	0,78197
	14	0,75179
	15	0,4995
Subset "HIGH *mTOR*" (set 1)		
	16	8,12868
	17	2,8871
	18	16,4435
	19	7,55925
	20	8,13857
	21	1,75096
	22	6,39349
	23	15,5318
	24	20,4619

25	19,5467
26	18,3461
27	2,34972
28	4,14626
29	1,93892
30	6,92782
31	2,40745
32	3,98337
33	2,68639
34	16,1098
35	1,83962
36	4,86948
37	1,78893
38	2,08107
39	2,94207
40	2,22511
41	2,28718
42	2,76822
43	9,54399
44	4,10254
45	6,13868
46	6,95261

	47	10,637
END-STAGE patients<br>(set 1)		
	48	30,3572
	49	27,4445
	50	11,4356
	51	8,01631
	52	1,73255
	53	1,85561
	54	7,7295
	55	1,95108
	56	4,17326
	57	8,48514
	58	6,98344
	59	11,8258
	60	5,13704
	61	19,9551
Subset<br>"LOW *mTOR*"<br>(set 2)		
	1	0,54626

	2	0,82674
	3	0,82808
	4	0,71773
	5	0,6904
	6	0,37478
	7	0,70077
	8	0,7754
Subset "HIGH *mTO*R" (set 2)		
	9	6,29006
	10	1,95969
	11	15,8527
	12	6,92144
	13	4,9694
	14	7,84087
	15	4,32442
	16	1,78696
	17	1,54719
	18	7,00695
END-STAGE patients (set 2)		

19	3,59205
20	4,43402
21	33,8368
22	1,91851
23	6,70406
24	1,962
25	17,569
26	3,73163
27	1,95205
28	1,58341
29	13,0912
30	6,04466
31	1,40992

Supplementary, Results

Clinical parameters of 18 OA outpatients (set 2)

Analysis of demographic and clinical characteristics of 18 OA outpatients (set 2) revealed that K&L OA grades of the examined subjects varied from 2 to 3 (grade II, 13 patients; grade III, 5 patients). The average disease duration was 14.0 years (range 2-29 years). The majority of patients exhibited Heberden's nodes and an increased BMI (range 27.2-39.1). All the patients had normal bone mineral density (BMD). The WOMAC score indicated moderate rates of knee pain in these OA outpatients. Synovitis at the knee joint was detected in 53% of the OA outpatients.

Whole blood gene expression

Examination of gene expression in the whole blood of 18 OA outpatients (set 2) revealed that *mTOR* was significantly downregulated in 8 patients compared to healthy controls, while 10 subjects exhibited *mTOR* gene upregulation (**Supplementary, Fig. 6A**). Therefore, all the examined OA outpatients were divided into 2 subsets: a "Low *mTOR*" expression subset (8 patients) and a "High *mTOR*" expression subset (10 patients). The data regarding relative *mTOR* gene expression is presented in **Supplementary, Table 3**.

Examination of the expression of *ULK1*, *p21*, *caspase 3*, and *TNFα* in the peripheral blood of set 2 OA outpatients demonstrated that the "Low *mTOR*" expression subset of OA outpatients exhibited significant upregulation of *TNFα* gene, while *ULK1, caspase 3,* and *p21* expression remained similar to that in healthy individuals (**Supplementary, Fig. 6B-E**). In contrast, the "High *mTOR*" expression subset of OA outpatients exhibited significant upregulation all of the examined genes in comparison to healthy controls (**Supplementary, Fig. 6)**. Gene expression studies in the 13 end-stage OA patients undergoing joint

replacement surgery (set 2) demonstrated that all of the examined genes were also overexpressed in their blood in comparison to healthy controls **(Supplementary, Fig. 6).**

Protein levels of phospho-p70-S6K in isolated PBMCs

We analyzed the protein levels of phospho-p70-S6K serine/threonine kinase in the PBMC fraction of whole blood from 18 OA outpatients (set 2) and 13 end-stage OA subjects (set 2). These studies showed that the "Low *mTOR*" expression subset OA outpatients possessed significantly lower phospho- p70-S6K protein concentrations in PBMCs compared to the "High *mTOR*" expression subset OA outpatients and to the end-stage OA subjects (**Supplementary, Fig. 6F**). When the amount of the examined proteins was evaluated and compared to amounts in healthy subjects, we observed that the "Low *mTOR*" subset OA outpatients possessed significantly lower phospho- p70-S6K protein. In contrast, protein concentrations of phospho-p70-S6K in the"High *mTOR*" subset OA outpatients and in the end-stage OA subjects significantly exceeded the concentrations in healthy individuals (**Supplementary, Fig. 6F**).

Examination of gene expression in the articular cartilage of the end-stage OA patients

Articular cartilage degradation in the end-stage OA patients at arthroplasty was associated with significantly higher levels of matrix metalloproteinases *MMP-13*, and *MMP-9,* as well as type X collagen (*COL10A1*) gene expression in the cartilage of another sample (set 2) of the end-stage OA patients compared to another sample of healthy subjects (set 2) (**Supplementary, Fig. 7**). This was accompanied by significant upregulation of *mTOR* expression and downregulation of *ULK1* in comparison to healthy controls (**Supplementary, Fig. 7).** At the same time, no significant changes in *caspase 3* and *p21* gene expression in these OA patients versus healthy subjects were observed.

Supplementary, FIGURE LEGENDS

Figure 5. A relationship between *mTOR* gene expression measured in the blood or articular cartilage from end-stage OA patients (n=11, set 2) without outliers (n=2, set 2).

Figure 6. Relative expression of the genes *mTOR* (A), *ULK1* (B), *p21* (C), *caspase 3* (D), and *TNFα* (E) with reference to *β-actin* determined by real-time PCR analyses of whole blood from "Low *mTOR*"subset (n=8), "High *mTOR*" subset (n=10) and end-stage OA patients (n=13) (set 2) compared with healthy controls (n=27); (F), protein concentrations of phospho-p70-S6K measured by ELISA in PBMCs from the "Low *mTOR*" subset (n=8), the "High *mTOR*" subset (n=10), and end-stage (n=13) OA patients (set 2), compared to control subjects (n=27). Asterisks indicate significant differences from the control in pairwise comparisons (Student's unpaired t-test).

Figure 7. Relative expression of the genes *mTOR, COL10A1, MMP-13, MMP-9* (A), and *ULK1, p21,* and *caspase 3* (B) with reference to *β-actin* as determined by real-time PCR analyses in osteoarthritic cartilage (n=13) (set 2) compared with healthy cartilage (n=12) (set 2). Controls are shown as 1.0 as required for relative quantification with the real-time PCR protocol. Asterisks indicate significant differences from the control (Student's unpaired t-test). Note: #, only one subject from a new set of cartilage samples from healthy individuals demonstrated MMP13 gene expression.

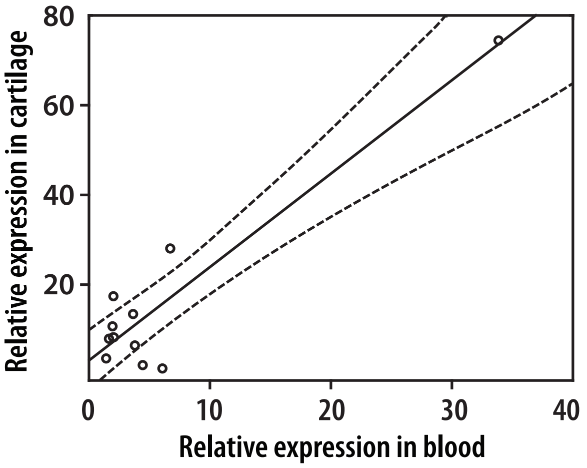
Relative expression in cartilage
80
60
40
20
0
10
20
30
40
Relative expression in blood

Figure 5

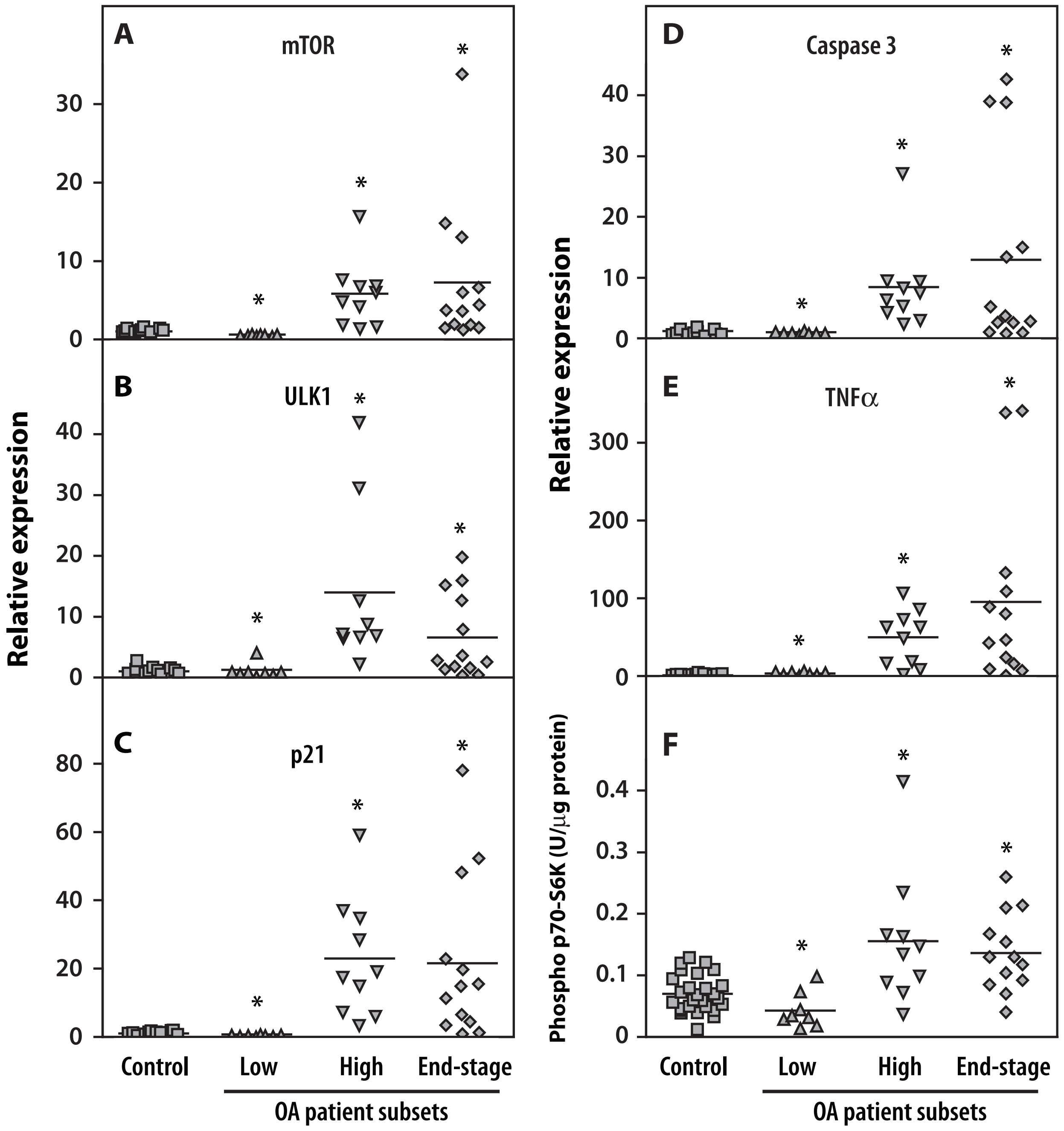
A
mTOR
B
ULK1
C
p21
D
Caspase 3
E
TNFα
F
Relative expression
Phospho p70-S6K (U/µg protein)
Control
Low
High
End-stage
OA patient subsets

Figure 6

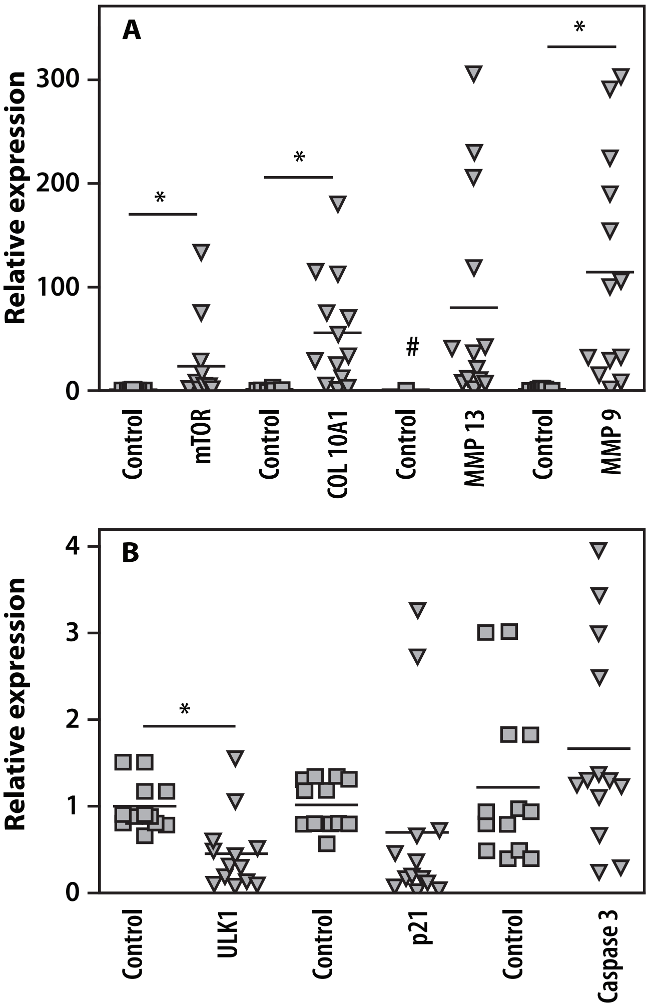
A
Relative expression
300
200
100
0
Control
mTOR
Control
COL 10A1
Control
MMP 13
Control
MMP 9
B
4
3
2
1
Control
ULK1
Control
p21
Control
Caspase 3

Figure 7
